# Mechanism and Kinetics of the Phase Formation and Dissolution of Na_x_WO_3_ on a Pt Electrode in a Na_2_WO_4_–WO_3_ Melt

**DOI:** 10.3390/ma16227207

**Published:** 2023-11-17

**Authors:** Alexander V. Kosov, Olga V. Grishenkova, Olga L. Semerikova, Sergey V. Vakarin, Yuriy P. Zaikov

**Affiliations:** Institute of High Temperature Electrochemistry, Ural Branch of the Russian Academy of Sciences, Yekaterinburg 620990, Russia; o.semerikova@ihte.ru (O.L.S.); s.vakarin@ihte.ru (S.V.V.); zaikov@ihte.ru (Y.P.Z.)

**Keywords:** oxide tungsten bronze, mathematical modeling, computer simulation, phase formation, mechanism, kinetics

## Abstract

A comprehensive study concerning the phase formation mechanism and growth/dissolution kinetics of sodium tungsten bronze crystals during the electrolysis of a 0.8Na_2_WO_4_–0.2WO_3_ melt was carried out. The regularities of deposit formation on a Pt(111) working electrode were investigated experimentally using cyclic voltammetry, chronoamperometry, scanning electron microscopy, and X-ray diffraction analysis. Models have been developed to calculate the current response during the formation, growth and dissolution of a two-phase deposit consisting of Na_x_WO_3_ and metallic tungsten or two oxide tungsten bronzes with different sodium content. These models consider mass transfer to the electrode and nuclei; chemical and electrochemical reactions with the participation of polytungstate ions, Na^+^, Na^0^, and O^2−^; as well as the ohmic drop effect. The approach was proposed to describe the dissolution of an Na_x_WO_3_ crystal with a nonuniform sodium distribution. The fitting of cyclic voltammograms was performed using the Levenberg–Marquardt algorithm. The Na_x_WO_3_ formation/growth/dissolution mechanism was determined. Concentration profiles and diffusion coefficients of [W_n_O_3n_]^−^, reaction rate constants, number density of nuclei, and time dependencies of crystal size were calculated. The proposed approaches and models can be used in other systems for the cyclic voltammogram analysis and study of the mechanism and kinetics of electrode processes complicated by phase formation; parallel and sequential electrochemical and chemical reactions; as well as the formation of a deposit characterized by a nonuniform phase and/or chemical composition.

## 1. Introduction

Oxide tungsten bronzes (OTBs) are nonstoichiometric compounds with the general formula M_x_WO_3_, where M is an element of the first or second groups of the periodic table, more often an alkali metal, and 0 < x < 1. Alkali metal ions are located inside channels formed by WO_6_ octahedra connected through a common bridging oxygen atom [1,2]. The symmetry of the OTB structure decreases as the value of x decreases, and the transition occurs from cubic (Figure S1) to tetragonal and hexagonal “tunnel” structures, which causes a cardinal change in the electrical, optical, and magnetic properties of M_x_WO_3_. Combinations of useful characteristics, including a wide range of electrophysical properties [3,4,5,6,7,8], a large color range, high corrosion resistance in water and acids, selectivity to certain cation types in aqueous solutions, etc. [9,10,11,12], allow the use of OTBs as electrodes in chemical analysis [12,13], sorbents [14,15], components of advanced materials [16,17,18,19,20] and anticorrosive coatings [21], and therapeutic agents [22]. OTB-based materials and OTB-containing hybrid systems are an inexpensive and highly efficient alternative in many important areas of catalysis and photocatalysis [23,24,25,26,27].

At present, there are many different methods for the synthesis of OTBs [24]. The electrochemical method based on the electrolysis of polytungstate melts has a number of advantages, including the low cost of the product, short synthesis time, nontoxicity (oxygen is released at the anode), and the ability to influence the composition and structure of OTBs by varying the electrolysis parameters [26,27,28,29,30,31]. However, the mechanism and kinetics of OTB electrodeposition are still poorly understood, which hinders effective control and production of OTBs with a given x value.

Various mechanisms for the formation and dissolution of OTBs in Na_2_WO_4_–WO_3_ melts were proposed based on the analysis of experimental dependencies obtained by electrochemical methods. In early works [30,31], it was suggested that the electroreduction of monomers (WO_3_), tetramers ((WO_3_)_4_) and (WO_3_)_18_ occurs at the cathode in electrolytes with a mole fraction of tungsten trioxide up to 0.15, from 0.25 to 0.50, and over 0.50, respectively. Fredlein and Damjanovic [32] noted the discrepancy between these conclusions and the actual OTB composition. To explain the formation of cubic OTB with x from 0.6 to 0.8, they assumed either subsequent sodium discharge or loss of WO_3_ from the lattice of a low-sodium bronze (or directly from [Na(WO_3_)_4_]_ad_) prior to incorporation into the lattice. Moreover, a hypothesis was put forward about simultaneous bronze dissolution and sodium dissolution from the deposit [32]. Randin [33,34] believed that the electrodeposition of sodium tungsten bronzes from melts containing 10, 20 and 50 mol% WO_3_ is a complex reaction controlled by at least three processes including diffusion in the electrolyte, heterogeneous reaction (adion surface diffusion), and charge transfer. Elwell et al. [35] proposed a model considering the resistances of these stages and concluded that the growth rate of OTB crystals is determined by both interfacial kinetics and diffusion in the melt, since the growth rate of a single OTB crystal increases if it rotates.

Later, high-temperature studies [36,37] proved the existence of tetrahedral anions (WO_4_)^2−^ in molten sodium tungstate and anionic chains (W_2_O_7_)^2−^ in molten sodium ditungstate. Taking into account these data, Baraboshkin and Kaliev [25,38] proposed various schemes for the formation of a cathode deposit with the participation of both simple (W^6+^) and bound into stable groups (${\mathrm{WO}}_{4}^{2-}$, $W_{2}O_{7}^{2-}$, $W_{3}O_{10}^{2-}$, $W_{4}O_{13}^{2-}$) tungsten-containing ions, and the lowest reduced forms (${\mathrm{WO}}_{n}^{(2n-5)-}$) soluble in the melt. Simulation results [29,39] indicated that the experimental facts cannot be explained without assuming the presence of more complex polymer groups ($W_{3}O_{10}^{2-}$, $W_{4}O_{13}^{2-}$, etc.) at a WO_3_ mole fraction above 0.2. Vorozhbit et al. [40] supported this conclusion but believed that the electroreduction of small sized and highly mobile alkali metal cations occurs primarily, and then the neutral atoms M^0^ diffuse into the diffusion layer and reduce the polymerized anionic groups.

Drobasheva and Spitsyn [28] considered polytungstate melts to be polysalts of alkali monotungstates and nonstoichiometric tungsten oxides with an intermediate oxidation state from five to six. However, this assumption was refuted in recent studies of the structure of molten M_2_O–WO_3_ mixtures (M = Na, K, Li) by high-temperature in situ Raman spectroscopy in combination with DFT analysis [41,42,43,44,45]. In these works, the formation of [WO_4_]^2−^, [W_2_O_7_]^2−^, [W_3_O_10_]^2−^, and [W_4_O_13_]^2−^ was only proved at M_2_O:WO_3_ ratios equal to 1:1, 1:2, 1:3, and 1:4, respectively.

Our comprehensive studies of the density and ionic equilibria in the Na_2_WO_4_–WO_3_ melt with a WO_3_ mole fraction up to 0.5 [46] indicate a more complex ionic composition and intricate dependencies of the equilibrium concentrations of Na^+^, ${\mathrm{WO}}_{4}^{2-}$, $W_{2}O_{7}^{2-}$, $W_{3}O_{10}^{2-}$, $W_{4}O_{13}^{2-}$, ${\mathrm{WO}}_{2}^{2+}$, and O^2–^ ions on the ratio of components in the initial oxide–salt mixture.

In this work, we present the results of studying the formation/growth/dissolution of a cathode deposit containing cubic OTB and the kinetics of processes occurring in the electrolyte, at the electrolyte/electrode interface, and at the electrolyte/new-phase interface during the electrolysis of the 0.8Na_2_WO_4_–0.2WO_3_ molten mixture at 1023 K. Another purpose of this work is the development of a general method for modeling cyclic voltammograms suitable for extracting quantitative information about the parameters of an electrochemical process complicated by the presence of stages of a different nature (electrochemical phase formation and chemical reactions in the electrolyte bulk and on interfaces).

## 2. Materials and Methods

Sodium tungstate and tungsten trioxide (purity 99.9 wt%, Vecton, St.-Petersburg, RF) were used to prepare the 0.8Na_2_WO_4_–0.2WO_3_ melt. The reagents were dried at 523 K for 2 h and then mixed into a porcelain container. The prepared mixture was melted in a platinum crucible.

The experiments were carried out at 1023 K in air. Autolab PGSTAT302N with Nova 1.9 software was used for electrochemical measurements and electrodeposition. Platinum foil with (111) texture was used as the working electrode (4 mm × 11 mm × 0.1 mm). The foil was preliminarily annealed at 973 K for 2 h to remove surface impurities. A similar piece of platinum foil semi-immersed in the melt under examination served as a reference electrode. The platinum crucible served as the auxiliary electrode. The melt temperature was measured with a Pt/Pt-Rh thermocouple and controlled using a Varta TP703 temperature controller (Varta, Moscow, Russia) with an accuracy of ±1 K.

The morphology and elemental composition of the samples synthesized under potentiostatic conditions were studied by scanning electron microscopy (SEM–EDS) using a TESCAN MIRA 3 LMU electron microscope (TESCAN, Brno, Czech Republic) with an INCA Energy 350 microanalysis system and an Oxford Instruments X–MAX80 energy dispersive spectrometer. To determine the phase composition of cathode products, X-ray diffraction analysis of the samples was carried out using a RIGAKU D/MAX-2200VL/PC X-ray diffractometer (Rigaku Corp., Tokyo, Japan) in CuK_α_ radiation. Before the studies, the samples were washed in an alkaline solution (10–15 wt % KOH) at room temperature for 12 h [47] and rinsed in distilled water and ethanol.

## 3. Experimental Results

### 3.1. Cyclic Voltammetry

**Figure 1:**
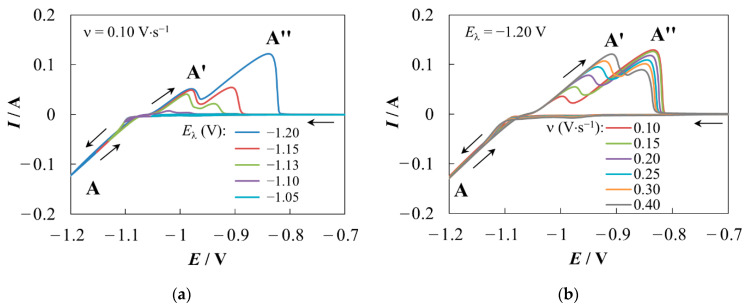
Effect of potential sweep parameters on CVs recorded using a Pt(111) working electrode in the 0.8Na_2_WO_4_–0.2WO_3_ melt at 1023 K. Variation of (**a**) reverse potential, *E*_λ_, or (**b**) scan rate, ν.

Typical cyclic voltammograms (CVs) obtained by varying the reverse potential, *E*_λ_ (V), and the scan rate, ν (V·s^−1^), are shown in Figure 1a,b, respectively.

At *E*_λ_ ≤ −1.05 V (Figure 1a), a current loop **A** is formed in the cathode part. Such nucleation loops are often recorded during the formation and growth of an electrode deposit on an indifferent electrode [48,49,50,51,52]. In this case, a significant increase in the cathode current is associated with the appearance and growth of supercritical new-phase nuclei. An increase in the electroactive area, due to the growth of the deposit up to the transition to the anodic region, leads to a higher absolute value of the cathode current during scanning in the reverse direction than in the forward one at the same potential value.

Taking into account the data from the literature on the overpotentials required for the formation of OTBs and their quasi-equilibrium potentials in Na_2_WO_4_–WO_3_ melts [29], the hypothesis about the connection between current loop **A** and the electrocrystallization of OTBs seems plausible. However, the following feature of loop **A** should be noted. A further shift of the reverse potential in the negative direction usually leads to a gradual transformation of the loop into a peak of a diffuse nature [52], which is not observed in this system even at *E*_λ_ of about −2 V. In addition, the behavior of the anodic peaks **A′** and **A″** attracts attention. The position of peak **A′** shifts in a positive direction and its amplitude increases as |*E*_λ_| or ν increases (see Figure 1). At the same time, the anodic peak **A″** demonstrates a decrease in amplitude as ν increases, which is typical for dissolution of the deposit [50,51]. Variation of the scan rate has little effect on the peak **A″** position, while the growth of |*E*_λ_| causes a noticeable positive shift of **A″**. The ratio of amplitudes **A′** and **A″** changes in favor of **A″** when ν decreases or |*E*_λ_| increases. There are various possible reasons for the above facts, including mixed mass transfer, the presence of several sequential or parallel electrode processes, and the nontrivial deposition/dissolution mechanism in this system.

### 3.2. Chronoamperometry

**Figure 2:**
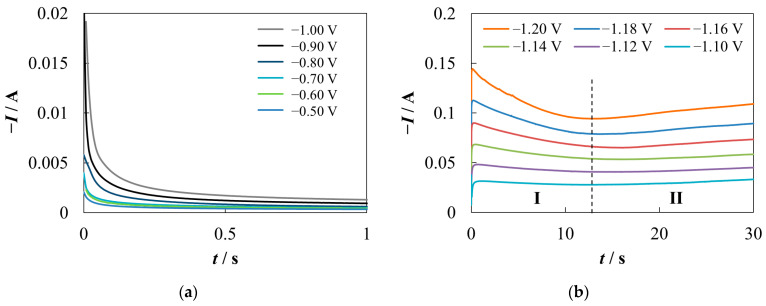
Time dependencies of the current in the 0.8Na_2_WO_4_–0.2WO_3_ melt on Pt(111) at 1023 K. Range of potentials: (**a**) −1.00 ≤ *E* ≤ −0.50 V; and (**b**) −1.20 ≤ *E* ≤ −1.00 V.

Figure 2 presents two typical chronoamperograms. In the range of potentials from −0.50 to −1.00 V (Figure 2a), the chronoamperograms demonstrate behavior similar to Cottrell’s dependence. Two regions can be conventionally distinguished on the chronoamperograms recorded at higher absolute values of the potential (Figure 2b).

The maximum and subsequent decrease in the cathode current can be seen in region **I** (see Figure 2b). The maximum is preceded by a minimum of the cathode current at *t* ≤ 0.06 s. An increase in |*E*| leads to an increase in |*I*| and a decrease in the time to reach extrema. This behavior is typical for nucleation/growth processes and is usually explained by an increase in the nuclei number density and the mutual influence of neighbors on the growth as the overpotential increases [53,54,55]. Some increase in the cathode current is observed in region **II**. The rise begins earlier the higher the |*E*| value.

**Figure 3:**
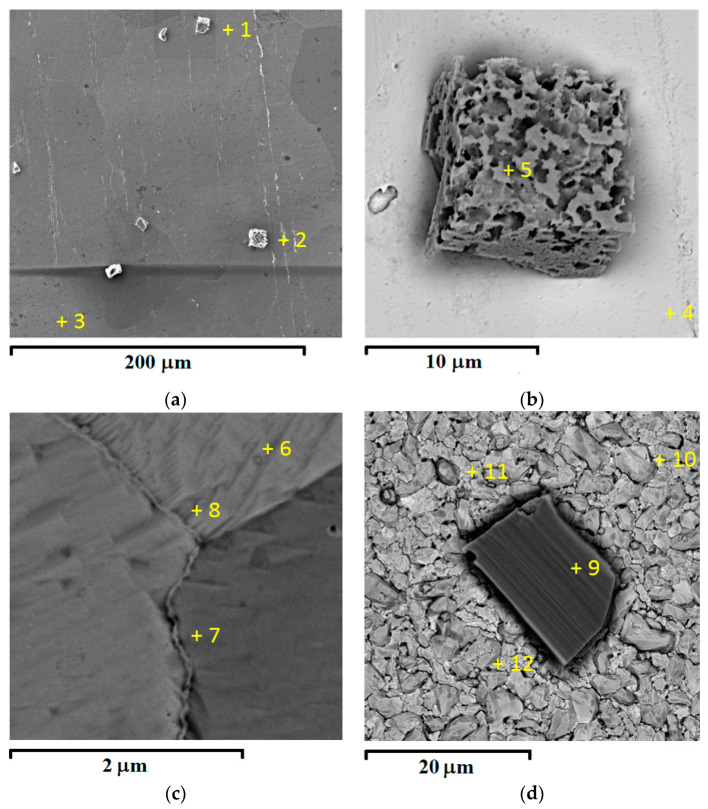
SEM images of an electrode surface area with the deposit formed during the potentiostatic electrolysis of the 0.8Na_2_WO_4_–0.2WO_3_ melt at 1023 K. Potential and electrolysis time values: (**a**–**c**) *E* = −1.05 V and *t* = 5 s; (**d**) *E* = −1.20 V and *t* = 60 s. The elemental composition is given in Table 1. Elemental maps for samples obtained under similar conditions are shown in Figures S2 and S3.

**Figure 4:**
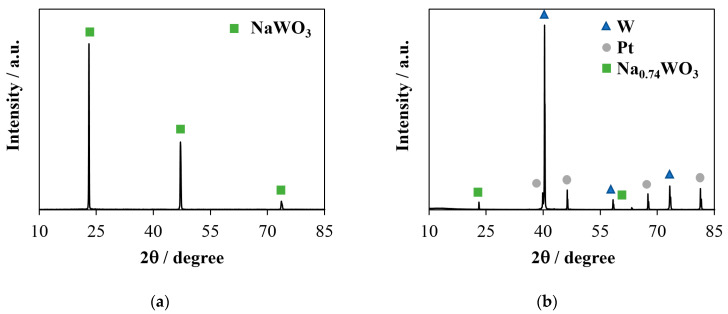
(**a**) XRD pattern for OTB crystals formed during the electrolysis of the 0.8Na_2_WO_4_–0.2WO_3_ melt at *T* = 1023 K and *E* = −1.05 V for 15 s; (**b**) XRD pattern of the sample shown in Figure 3d. The original XRD patterns are additionally provided in Figures S4 and S5.

The SEM-EDS (Figure 3 and Table 1) and XRD (Figure 4) data confirm that the change in the shape of the chronoamperograms at *E* ≤ −1.05 V is indeed associated with the formation of a cathode deposit.

At an electrodeposition time of about 5–10 s, the deposit predominantly consists of cubic crystals (Figure 3a,b) with the isostructural formula NaWO_3_ (Figure 4a). In addition, trace amounts of tungsten are also found on the surface of the platinum substrate (Figure 3c). An increase in the electrolysis time leads to a noticeable increase in the proportion of metallic tungsten in the deposit and the degree of electrode coverage with the deposit (Figure 3d and Figure 4b), which can explain the behavior seen in the chronoamperograms in region **II**. Figure 3 and Figure 4 also show that an increase in the absolute value of the potential promotes an increase in the average size and number of OTB and tungsten crystals, as well as a decrease in the sodium content in bronze.

A number of models have been developed which suggest analytical expressions for the analysis of the initial stages of electrochemical phase formation uncomplicated by any other chemical/electrochemical processes in cases of diffusion [53,56], kinetic [55,57], and mixed (diffusion + charge transfer) [58,59,60] growth control. Obviously the mechanism of deposit formation in our case is more complex, so none of these models can be directly used for treatment of chronoamperograms. Nevertheless, a comparison of the shape of *I*(*t*) dependencies in Figure 3b with [53,61] apparently allows us to exclude the hypothesis of purely diffusion controlled growth. In addition, the low filling of the substrate with the deposit at *t* close to the time corresponding to the current maximum contradicts the theory of growth controlled by the discharge of depositing ions under potentiostatic conditions [53,57].

Comparison with works investigating the influence of various factors (multi-stage process and mixed growth control [59,60,62,63], growth controlled by diffusion in a layer of finite thickness [64,65,66], and the migration effect [67,68,69]) on the current response under potentiostatic or potentiodynamic conditions indicates the need to consider combinations of these effects. Therefore, we used numerical simulation for a more detailed study of the mechanism and kinetics of deposit formation/dissolution in this system.

## 4. Models

### 4.1. Deposit Consisting of OTB and Tungsten

Let us assume that the formation of OTB can be realized due to the occurrence of both electrochemical processes involving ${\mathrm{WO}}_{2}^{2+}$, $W_{n}O_{3n+1}^{2-}$ and Na^+^ ions and chemical processes involving ${\mathrm{WO}}_{2}^{+}$, $W_{n}O_{3n}^{-}$ and Na^0^, while the formation of metallic tungsten occurs as a result of electroreduction of ${\mathrm{WO}}_{2}^{2+}$, ${\mathrm{WO}}_{2}^{+}$ and $W_{n}O_{3n}^{-}$. Considering the most probable interactions between these particles, as well as ${\mathrm{WO}}_{4}^{2-}$ and ${\mathrm{WO}}_{2}^{2+}$ ions [46], we obtain the following set of reactions:

(1) $${\mathrm{WO}}_{4}^{2-}\overset{k_{f}}{\underset{k_{b}^{*}}{⇄}}{\;\mathrm{WO}}_{2}^{2+}+2O^{2-}\;\;\mathrm{in}\;\mathrm{the}\;\mathrm{melt},$$

(2) $${\mathrm{WO}}_{2}^{2+}+\;1e\;\overset{k_{f}}{\underset{k_{b}}{⇄}}{\;\mathrm{WO}}_{2}^{+}\;\;\mathrm{on}\;\mathrm{Pt},$$

(3) $${\mathrm{WO}}_{2}^{2+}+\;6e\;\overset{k_{f}}{\underset{k_{b}^{*}}{⇄}}{\;W}_{\left(s\right)}\;+\;2O_{}^{2-}\;\;\mathrm{on}\;W,$$

(4) $${\mathrm{WO}}_{2}^{+}+\;5e\;\overset{k_{f}}{\underset{k_{b}^{*}}{⇄}}{\;W}_{\left(s\right)}\;+\;2O_{}^{2-}\;\;\mathrm{on}\;W,$$

(5) $${\mathrm{WO}}_{2}^{2+}+\;1e+W_{n}O_{3n+1}^{2-}+\;{\mathrm{Na}}^{+}\;\overset{k_{f}^{*}}{\underset{k_{b}^{}}{⇄}}{\;(n+1)\mathrm{Na}}_{x}{\mathrm{WO}}_{3\;(s)}\;\;\mathrm{on}\;\mathrm{OTB},\;x=1/(n+1),$$

(6) $${\mathrm{WO}}_{2}^{+}+W_{n}O_{3n+1}^{2-}+{\mathrm{Na}}^{+}\;\overset{k_{f}^{*}}{\underset{k_{b}^{}}{⇄}}{\;(n+1)\mathrm{Na}}_{x}{\mathrm{WO}}_{3\;(s)}\;\;\mathrm{on}\;\mathrm{OTB},\;x=1/(n+1),$$

(7) $${\mathrm{Na}}^{+}+1e+W_{n}O_{3n+1}^{2-}\;\overset{k_{f}^{*}}{\underset{k_{b}^{*}}{⇄}}{\;\mathrm{nNa}}_{x}{\mathrm{WO}}_{3\;(s)}{\;+\;O}^{2}{}^{-}\;\;\mathrm{on}\;\mathrm{OTB},\;x=1/n,$$

(8) $$W_{n}O_{3n}^{-}+{\mathrm{Na}}^{+}\;\overset{k_{f}^{*}}{\underset{k_{b}^{}}{⇄}}{\;\mathrm{nNa}}_{x}{\mathrm{WO}}_{3\;(s)}\;\;\mathrm{on}\;\mathrm{OTB},\;x=1/n,$$

(9) $${\mathrm{Na}}^{0}+W_{n}O_{3n+1}^{2-}\;\overset{k_{f}^{*}}{\underset{k_{b}^{*}}{⇄}}{\;\mathrm{nNa}}_{x}{\mathrm{WO}}_{3\;(s)}{\;+\;O}^{2}{}^{-}\;\;\mathrm{on}\;\mathrm{OTB},\;x=1/n,$$

(10) $${\mathrm{Na}}^{+}\;+\;1e\;\overset{k_{f}}{\underset{k_{b}^{}}{⇄}}{\;\mathrm{Na}}^{0}\;\;\mathrm{on}\;\mathrm{Pt},$$

(11) $$W_{n}O_{3n+1}^{2-}+1e\;\overset{k_{f}}{\underset{k_{b}^{*}}{⇄}}{\;W}_{n}O_{3n}^{-}\;+\;O_{}^{2-}\;\;\mathrm{on}\;\mathrm{Pt},$$

(12) $${\mathrm{Na}}^{0}+W_{n}O_{3n+1}^{2-}\;\overset{k_{f}^{*}}{\underset{k_{b}^{*}}{⇄}}{\;\mathrm{Na}}^{+}+{\mathrm{WO}}_{2}^{+}+W_{n-1}O_{3n-2}^{2-}+O^{2-}\;\;\mathrm{in}\;\mathrm{the}\;\mathrm{melt}.$$

**Figure 5:**
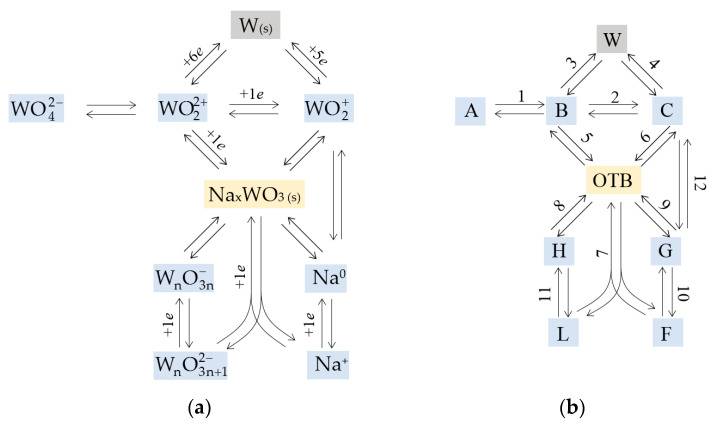
Scheme of the supposed chemical and electrochemical reactions in the normal (**a**) and formalized (**b**) form. Reagents present in the melt are highlighted in blue. Two solid products (tungsten and OTB) are highlighted in gray and yellow, respectively. Symbols and reaction numbers from the formalized scheme are used in the equations below.

The set of these reactions can be represented in the form of a scheme shown in Figure 5a. To write the equations, we formalize this scheme (Figure 5b).

The concentrations of polytungstate anions and Na^+^ ions are much higher than those of tungsten-containing cations, and oxide ions participate in many equilibria [46], so the concentrations of A, F, L and O^2−^ can be considered constant. In this case, the diffusion problem for the remaining reactants present in the melt (B, C, G and H) is described by the following system of equations:

(13) $$\left\{\begin{array}{l} \frac{\partial \;c_{B}}{\partial \;t}=k_{f1}c_{A}-k_{b1}^{*}c_{B}+D_{B}\frac{\partial^{2}c_{B}}{\partial \xi^{2}}\\ \frac{\partial \;c_{C}}{\partial \;t}=k_{f12}^{*}c_{G}-k_{b12}^{*}c_{C}+D_{C}\frac{\partial^{2}c_{C}}{\partial \xi^{2}}\\ \frac{\partial \;c_{G}}{\partial \;t}=-k_{f12}^{*}c_{G}+k_{b12}^{*}c_{C}+D_{G}\frac{\partial^{2}c_{G}}{\partial \xi^{2}}\\ \frac{\partial \;c_{H}}{\partial \;t}=D_{H}\frac{\partial^{2}c_{H}}{\partial \xi^{2}} \end{array}\right.,$$

where *c* ≡ *c*(ξ,*t*) (cm^−3^) and *D* (cm^2^s^−1^) are the concentration and diffusion coefficient of the indicated reagent, respectively, ξ (cm) is the coordinate, *t* (s) is the time, and *k*_f_ and *k*_b_ (s^−1^) are the rate constants of the forward and backward processes, respectively. The symbol * is used for rate constants adjusted due to the fact that not only A, B, C and G are involved in the forward and/or reverse process:

(14) $$k_{b1}^{*}=k_{b1}^{}c_{O^{2-}}^{2},\;\;\;k_{f12}^{*}=k_{f12}^{}c_{W_{n}O_{3n+1}^{2-}}^{},\;\;\;k_{b12}^{*}=k_{b12}^{}c_{{\mathrm{Na}}^{+}}c_{W_{n-1}O_{3n-2}^{2-}}^{}c_{O^{2-}}.$$

The equations in (13) do not contain migration terms due to the assumption that the concentrations A and F are constant. This approach allows us to avoid the significant mathematical and computational challenges associated with solving the Nernst–Planck–Poisson problem [64,65,66]. Nevertheless, the effect of a high ion concentration will be taken into account below via ohmic potential drop. The correctness of this method was proven in [70] for individual, i.e., not containing supporting electrolyte, salt melts.

To derive the boundary conditions for ξ = 0, we take into account the following points. For an arbitrary electrochemical reaction X + *ze* ⇄ Y, the flux, $J=-J_{X}^{}=J_{Y}^{}$ (s^–1^), on a surface of area *S* (cm^2^) can be written as [71,72]:

(15) $$J=S(k_{f}^{s}c_{X}^{s}-k_{b}^{s}c_{Y}^{s}),$$

(16) J=I/ze,

(17) $$k_{f}^{s}=K_{f}\exp(\alpha f\eta ),\;k_{b}^{s}=K_{b}\exp(-\beta f\eta ),$$

where $k_{f}^{s}$ and $k_{b}^{s}$ (cm·s^−1^) are the rate constants of the forward and backward processes occurring at the electrolyte/solid phase interface; *c*^s^ (cm^−3^) is the surface concentration; *I* (A) is the current; *z* is the number of electrons involved in the electrochemical reaction; *e* (C) is the elementary electric charge; *K*_f_ and *K*_b_ are pre-exponential factors, which are conveniently written as $K_{f}=k_{0}/{\left(c_{X}^{s}\right)}_{0}$ and $K_{b}=k_{0}/{\left(c_{Y}^{s}\right)}_{0}$ if X and Y are components of the electrolyte; *k*_0_ (cm^−2^s^−1^) is the heterogeneous rate constant; (*c*^s^)_0_ (cm^−3^) is the equilibrium (initial) value of the surface concentration; α and β are the charge transfer coefficients; α + β = 1, f=ze/kT, *k* (J·K^−1^) is the Boltzmann constant; *T* (K) is the absolute temperature; η (V) is the overpotential; η = *E*_0_ – *E*, *E*_0_ is the equilibrium potential, i.e., the potential value established after an exposure of the electrode in the electrolyte without applying current or voltage; and *E* is the electrode potential. In our case, the melt does not contain a supporting electrolyte (i.e., in this sense, it can be considered to be an individual salt melt), so the ohmic drop, η_Ω_ (V), should be taken into account in (17) [70]:

(18) $$k_{f}^{s}=K_{f}\exp[\;\alpha f(\eta -\eta_{\Omega })],\;k_{b}^{s}=K_{b}\exp[\;\beta f(\eta_{\Omega }-\eta )],$$

(19) $$\eta_{\Omega }=\left\{\begin{array}{l} I\;R\;\;\mathrm{for}\;\mathrm{reaction}\;\mathrm{on}\;\mathrm{the}\;\mathrm{electrode}\;\mathrm{surface},\;\\ \frac{I_{\Sigma }}{2\pi \;rN\;\kappa }\;\;\mathrm{for}\;\mathrm{reaction}\;\mathrm{on}\;\mathrm{the}\;\mathrm{deposit}\;\mathrm{surface}, \end{array}\right.$$

where *R* (Ω) is the cell resistance; *I*_Σ_ (A) is the total current to all new-phase nuclei; *r* (cm) is the radius of the nucleus approximated by a hemisphere (we assume that all nuclei have the same size); *N* is the number of nuclei; and κ (S·cm^−1^) is the specific electrical conductivity. Equations (15) and (17) are also applicable for a chemical reaction X ⇄ Y occurring at the liquid/solid interface if *z* = 0, i.e., exp(α*f*η) = exp(−β*f*η) = 1.

The diffusion flux of a reagent on the surface is equal to the algebraic sum of the fluxes associated with an increase or decrease in its surface concentration (due to participation in reactions at the interface) [71]. Thus, we obtain the following boundary conditions for B, C, G and H at ξ = 0:

(20) $$-D_{B}{\left.\frac{\partial \;c_{B}}{\partial \;\xi }\right|}_{\xi =0}+(k_{f2}^{s}+pk_{f3}^{s}+qk_{f5}^{s*})c_{B}^{s}-k_{b2}^{s}c_{C}^{s}-pk_{b3}^{s*}-qk_{b5}^{s}=0,$$

(21) $$-D_{C}{\left.\frac{\partial \;c_{C}}{\partial \;\xi }\right|}_{\xi =0}+(k_{b2}^{s}+pk_{f4}^{s}+qk_{f6}^{s*})c_{C}^{s}-k_{f2}^{s}c_{B}^{s}-pk_{b4}^{s*}-qk_{b6}^{s}=0,$$

(22) $$-D_{G}{\left.\frac{\partial \;c_{G}}{\partial \;\xi }\right|}_{\xi =0}+(qk_{f9}^{s*}+k_{b10}^{s*})c_{G}^{s}-qk_{b9}^{s*}-k_{f10}^{s}c_{F}^{s}=0,$$

(23) $$-D_{H}{\left.\frac{\partial \;c_{H}}{\partial \;\xi }\right|}_{\xi =0}+(qk_{f8}^{s*}+k_{b11}^{s})c_{H}^{s}-qk_{b8}^{s}-k_{f11}^{s}c_{L}^{s}=0,$$

where

(24) $$p=S_{W}/S_{e},\;q=S_{\mathrm{OTB}}/S_{e},$$

(25) $$\begin{array}{l} k_{b3}^{s*}=k_{b3}^{s}c_{O^{2-}}^{2},\;k_{b4}^{s*}=k_{b4}^{s}c_{O^{2-}}^{2},\;k_{f5}^{s*}=k_{f5}^{s}c_{{\mathrm{Na}}^{+}}c_{W_{n}O_{3n+1}^{2-}}^{},\\ k_{f6}^{s*}=k_{f6}^{s}c_{{\mathrm{Na}}^{+}}c_{W_{n}O_{3n+1}^{2-}}^{},\;k_{f7}^{s*}=k_{f7}^{s}c_{W_{n}O_{3n+1}^{2-}}^{},\;k_{f8}^{s*}=k_{f8}^{s}c_{{\mathrm{Na}}^{+}}\\ k_{f9}^{s*}=k_{f9}^{s}c_{W_{n}O_{3n+1}^{2-}}^{},\;k_{b9}^{s*}=k_{b9}^{s}c_{O^{2-}}^{},\;k_{b10}^{s*}=k_{b10}^{s}c_{O^{2-}}^{} \end{array},$$

*S*_W_ and *S*_OTB_ (cm^2^) are the electroactive areas of the tungsten and OTB, respectively, and *S*_e_ (cm^2^) is the electrode area. Equations (20)–(25) consider that $c_{W}^{s}=c_{\mathrm{OTB}}^{s}=1$.

Conditions (20)–(23) must be supplemented with the second boundary condition (at ξ = δ, where δ is the diffusion layer thickness) and the initial one (at *t* = 0). For B initially present in the melt, we have

*c*(δ,*t*) = *c*_0_, (26)

*c*(ξ,0) = *c*_0_, (27)

where *c*_0_ (cm^–3^) is the bulk concentration. For C, G and H, we will assume that

*c*(δ,*t*) = 0, (28)

*c*(ξ,0) = (*c***^s^**)_0_ − (*c***^s^**)_0_ξ/δ. (29)

Let us now consider the phase formation in this system. In this work, we will simulate the simplest case, when all nuclei of one phase appear simultaneously, have the same size, and do not overlap. This is a common approximation that can describe the initial stages of electrocrystallization of nuclei formed on an indifferent electrode in a short (compared to the entire time scale of the experiment) time interval. The formation of stable nuclei of new phases (W and OTB) on the electrode surface is realized when the algebraic sum of the substance flow to the nuclei is positive:

(30) $$k_{f3}^{s}c_{B}^{s}+k_{f4}^{s}c_{C}^{s}-k_{b3}^{s*}-k_{b4}^{s*}>0,$$

(31) $$k_{f5}^{s*}c_{B}^{s}+k_{f6}^{s*}c_{C}^{s}+k_{f7}^{s}c_{F}^{s}c_{L}^{s}+k_{f8}^{s*}c_{G}^{s}+k_{f9}^{s*}c_{H}^{s}-k_{b5}^{s}-k_{b6}^{s}-k_{b7}^{s}-k_{b8}^{s}-k_{b9}^{s}>0.$$

If expressions (30) and (31) are valid then the growth rate exceeds the dissolution rate and the increment in the nucleus volume, *V* (cm^3^), due to the attachment of *g* new-phase particles can be determined as follows:

(32) dV=υ dg,

(33) $$dg=\left(\sum_{m}j_{m}\right)S_{\mathrm{nuc}}\;dt,$$

where υ(cm^3^) is the volume of one new-phase atom (for W) or the average volume of one attached particle (for OTB); *j*_m_ (cm^–2^s^–1^) is the flux density to the nucleus due to reactions (3) and (4) for W and (5)–(8) for OTB; and *S*_nuc_ (cm^2^) is the surface area of the nucleus,

(34) $$S_{\mathrm{nuc}\;W}=2\pi r^{2}={\left(18\pi \right)}^{1/3}V^{2/3},$$

(35) $$S_{\mathrm{nuc}\mathrm{OTB}}=5a^{2}=5\;V^{2/3},$$

(36) $$S_{\mathrm{nuc}W}=S_{W}/N_{W},\;S_{\mathrm{nuc}\mathrm{OTB}}=S_{\mathrm{OTB}}/N_{\mathrm{OTB}}.$$

Here *a* (cm) and *r* (cm) are the length of the cube edge for the cubic OTB nucleus and the radius for the hemispheric tungsten nucleus, respectively; and *N*_W_ and *N*_OTB_ are the number of W and OTB nuclei, respectively. The equation can be derived from (32)–(35) for calculating the change in the nucleus volume:

(37) $$\frac{dV}{dt}=b\upsilon V^{2/3}\;\sum_{m}j_{m},$$

where $b={\left(18\pi \right)}^{1/3}$ for W and *b* = 5 for OTB.

### 4.2. Deposit Containing Two OTBs

Experimental data show that the proportion of tungsten in the deposit is negligible at a short deposition time, and the sodium content in OTB can be lower at a higher cathodic potential. Therefore, we will also consider the case when the cathode deposit consists of two OTBs which differ in their growth/dissolution kinetics and, hence, in their x value. This leads to a modification of the scheme shown in Figure 5, namely, reactions (3) and (4) should be deleted, and reactions (5)–(9) should be duplicated by similar ones characterized by other rate constants. Accordingly, the boundary conditions for B, C, G and H at ξ = 0 should be replaced by the following:

(38) $$-D_{B}{\left.\frac{\partial \;c_{B}}{\partial \;\xi }\right|}_{\xi =0}+(k_{f2}^{s}+qk_{f5}^{s}+q\prime k_{f5}^{s*}\prime )c_{B}^{s}-k_{b2}^{s}c_{C}^{s}-qk_{b5}^{s*}-q\prime k_{b5}^{s}\prime =0,$$

(39) $$-D_{C}{\left.\frac{\partial \;c_{C}}{\partial \;\xi }\right|}_{\xi =0}+(k_{b2}^{s}+qk_{f6}^{s}+q\prime k_{f6}^{s*}\prime )c_{C}^{s}-k_{f2}^{s}c_{B}^{s}-qk_{b6}^{s*}-q\prime k_{b6}^{s}\prime =0,$$

(40) $$-D_{G}{\left.\frac{\partial \;c_{G}}{\partial \;\xi }\right|}_{\xi =0}+(qk_{f9}^{s*}+q\prime k_{f9}^{s*}\prime +k_{b10}^{s*})c_{G}^{s}-qk_{b9}^{s*}-q\prime k_{b9}^{s*}\prime -k_{f10}^{s}c_{F}^{s}=0,$$

(41) $$-D_{H}{\left.\frac{\partial \;c_{H}}{\partial \;\xi }\right|}_{\xi =0}+(qk_{f8}^{s*}+q\prime k_{f8}^{s*}\prime +k_{b11}^{s})c_{H}^{s}-qk_{b8}^{s}-q\prime k_{b8}^{s}\prime -k_{f11}^{s}c_{L}^{s}=0.$$

All terms of Equations (38)–(41) related to the second OTB are marked with an apostrophe.

### 4.3. Inhomogeneity of Sodium Distribution in an OTB Crystal

The structure of OTB crystals determines the ability of sodium to move inside the channels formed by WO_6_ octahedra [1]. This allows us to suspect the inhomogeneity of the sodium distribution in the crystal associated with the formation of an internal region with a constant x value, which differs from that in the surface layer. In the intermediate region, the change in the x value can be approximately described by the equation

(42) $$x=wx^{\mathrm{up}}+(1-w)x^{\mathrm{in}},\;w=0.5\left\{\mathrm{erf}\left[\frac{4(\zeta -\zeta_{0})}{\Delta \zeta }\right]+1\right\},$$

where *w* is the weight coefficient; x^up^ and x^in^ are the sodium content in the upper (surface) layer and inside the crystal, respectively; ζ and ζ_0_ (cm) are the distances from the center of the crystal base to a given point inside the crystal and to the interface between the two regions, respectively; and Δζ is the thickness of the transition layer. Equation (42) is a solution to the diffusion problem for the contact of two solutions with different concentrations. The rate constants for reactions (5)–(8) are related to x, so we will use similar equations during the dissolution of crystals:

(43) $$K_{f}=wK_{f}^{\mathrm{up}}+(1-w)K_{f}^{\mathrm{in}},$$

(44) $$K_{b}=wK_{b}^{\mathrm{up}}+(1-w)K_{b}^{\mathrm{in}}.$$

Thus, the dissimilarity between x^up^ and x^in^ will lead to differences in the dissolution rates of the above crystal regions.

### 4.4. Parameters and Simulation Procedure

To simulate voltammograms in accordance with the model described in Section 4.1, the system of Equations (13)–(15), (17)–(31), (36) and (37) was numerically solved taking into account the time dependence of the overpotential,

(45) $$\eta =\left\{\begin{array}{l} \nu t\;,\;0\leq t<t_{\lambda }\\ \nu (2t_{\lambda }-t)\;,\;t\geq t_{\lambda } \end{array}\right.,$$

where ν (V·s^−1^) is the scan rate, and *t*_λ_ (s) is the reverse time. Expressions (20)–(23) were replaced by Equations (38)–(41) to simulate the current response in the case of the formation and dissolution of two OTBs. Formulas (42)–(44) were added to this system of equations to simulate the complicated mechanism of the dissolution of OTB crystals.

The following parameter values were used in the calculations: *z*_3_ = 6; *z*_4_ = 5; *z*_2_ = *z*_5_ =*z*_7_ = *z*_10_ = *z*_11_ =1; *z*_1_ = *z*_6_ =*z*_8_ = *z*_9_ = *z*_12_ = 0; α = β = 0.5; υ_W_ = 1.6 × 10^−23^ cm^3^; υ_OTB_ = 5.7 × 10^−23^ cm^3^; *R* = 0.35 Ω; κ = 0.9 S·cm^−1^; δ = 0.02 cm; *S*_e_ = 0.88 cm^2^; *T* = 1023 K; *E*_0_ = 0 V; η_λ_ from 1.13 to 1.20 V; and ν from 0.10 to 0.40 V·s^−1^. The υ_OTB_ value was determined by taking into account the lattice parameter calculated using the Brown–Banks equation [73]. The initial value of the number density of nuclei, *N*_0_ = 1.24 × 10^4^ cm^−2^, was estimated from the experimental data. The initial values of the diffusion coefficients for B, C, G and H were taken as equal to *D*_B_ = *D*_C_ = *D*_G_ = *D*_H_ = 2 × 10^−5^ cm^2^s^−1^. The initial concentrations of all ions, *c*_0_ (cm^−3^), were taken from Table 2 in our previous work [46]. The initial values of the remaining parameters were chosen arbitrarily.

The backward Euler method was used in mass transfer modeling to ensure the stability and convergence of the solution. The forward Euler method was applied to calculate the nucleus volume using Equation (37). The finite difference code was implemented in Excel using the built-in VBA programming language [74]. The fitting of unknown parameters was performed according to the Levenberg–Marquardt algorithm.

## 5. Simulation Results and Discussion

**Figure 6:**
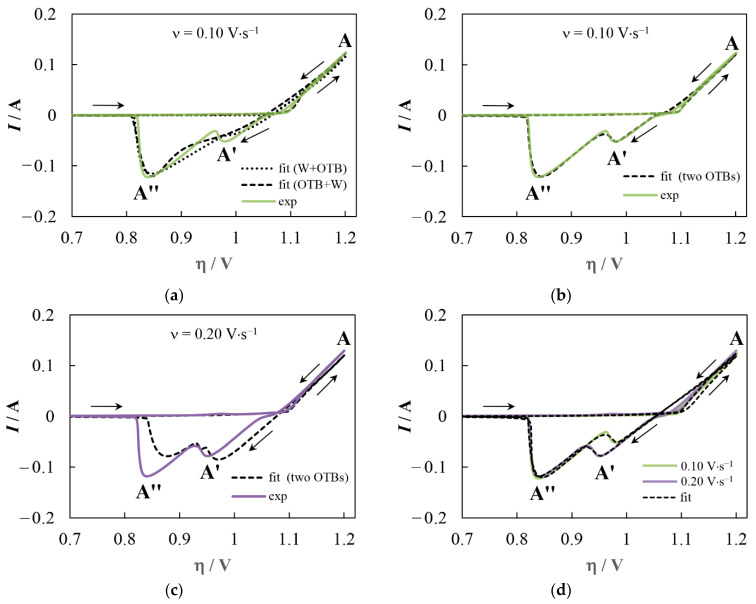
Comparison of experimental and fitted CVs for deposits of different phase compositions. The deposit consists of (**a**) OTB and tungsten (see Section 4.1); (**b**,**c**) two OTBs (see Section 4.2); (**d**) OTB with a sodium-depleted surface layer (see Section 4.3). For (**a**) the dotted line is calculated for the case where peak A′ is related to tungsten dissolution and peak A″ is related to OTB dissolution, while the dashed line corresponds to the opposite case.

Figure 6 shows a comparison of experimental and fitted CVs for the formation/dissolution of deposits with different phase compositions. For a deposit consisting of tungsten and OTB, two options were simulated (Figure 6a). A good match was not achieved, regardless of whether the **A′** peak was attributed to the tungsten dissolution or the OTB dissolution. When simulating the growth and dissolution of the two OTBs, a fairly good fit was observed under some conditions (Figure 6b), but it was easily broken by varying the scan parameters (Figure 6c).

Excellent agreement between the anodic branches of the experimental and fitted curves was obtained by simulating the dissolution of a single-phase deposit consisting of Na_x_WO_3_ crystals with a nonuniform distribution of sodium (Figure 6d). In this case, peaks **A′** and **A**″ are associated with the dissolution of the surface layer and the inner region of Na_x_WO_3_ crystals, respectively. However, the calculated nucleation loop is always wider than the experimental one in this case, and the inflection at the transition to the anode region is not reproduced.

**Figure 7:**
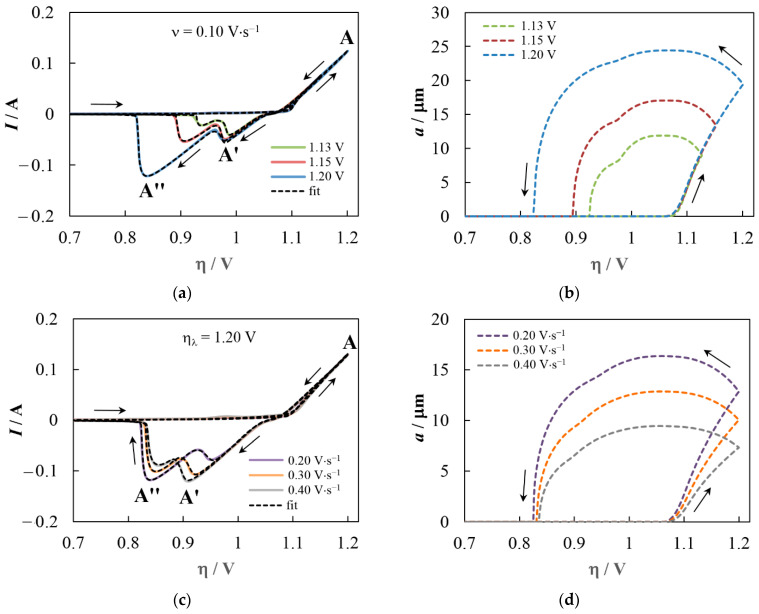
(**a**) The results of fitting a set of CVs at different reverse overpotentials and (**b**) the corresponding calculated overpotential dependencies of the OTB crystal size; (**c**) The results of fitting a set of CVs at different scan rates; and (**d**) the corresponding calculated overpotential dependencies of the OTB crystal size. The dissolution process is simulated according to the model described in Section 4.3. The coefficient of determination, *R*^2^, is at least 0.998 for any CV.

The best fitting of both the anode and cathode branches of CVs can be performed for the growth and dissolution of OTB crystals with a nonuniform distribution of sodium (Figure 7a,c) when the pre-exponential factors for the backward reactions change smoothly near the crossover overpotential η_0_ in the range Δη:

(46) $$K_{b}=wK_{b}^{c}+(1-w)K_{b}^{a},\;w=0.5\left\{\mathrm{erf}\left[\frac{4(\eta -\eta_{0})}{\Delta \eta }\right]+1\right\}.$$

Here, the superscripts “c” and “a” refer to the cathode and anode branches of the CV, respectively; η_0_ = 1.067 V and Δη = 0.091 V. In practice, the above variation can be associated with a change in the composition of the OTB surface layer at decreasing overpotential or with passivation of the crystal surface by impurities.

**Figure 8:**
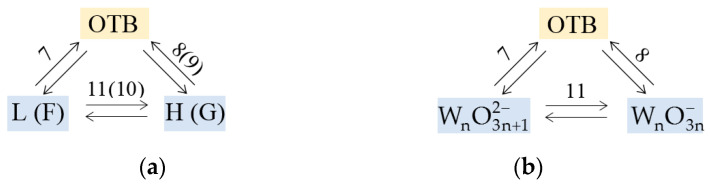
(**a**) Combined and (**b**) final schemes of OTB formation providing optimal fitting.

The fitting shows that reactions (5), (6) and (12) can also be excluded, in addition to reactions (3) and (4) which are associated with the formation of tungsten, since their contribution to the current response is negligible. Thus, the mechanism can be described by a simpler scheme including reactions (7)–(11). The L-H-OTB branch is similar to the F-G-OTB one (see Figure 5), because L and F are high-concentration oxidized forms, H and G are reduced forms, *z* = 1 in reactions (10) and (11), and *z* = 0 in reactions (8) and (9). Therefore, we can use the simplest scheme for fitting, in which reactions (10) and (11), and (8) and (9) are combined (Figure 8a).

The fitting results using scheme on Figure 8a show that the equilibrium concentration of the reduced form is (2.8 ± 0.1) × 10^13^ cm^–3^. If the OTB is formed mainly due to reactions (7), (9) and (10), then the standard potential for reaction (10),

(47) $$E^{0}=\frac{1}{f}\ln\frac{{\left(c_{{\mathrm{Na}}^{+}}^{s}\right)}_{0}}{{\left(c_{{\mathrm{Na}}^{0}}^{s}\right)}_{0}},$$

is equal to −1.76 V. However, the standard potential calculated for reaction

(48) $${\mathrm{Na}}^{0}+{\mathrm{WO}}_{3}+0.5O_{2\;(g)}={\mathrm{Na}}_{2}{\mathrm{WO}}_{4}$$

using the HSC software [75] is −3.03 V. Therefore, the contribution of reactions (9) and (10) can be ignored.

The simplest final scheme (Figure 8b) assumes the growth of Na_x_WO_3_ crystals due to reactions (7) and (8). The parameter values providing the best match (similar to that shown in Figure 7a,c) according to scheme on Figure 8b are shown in Table 2.

**Figure 9:**
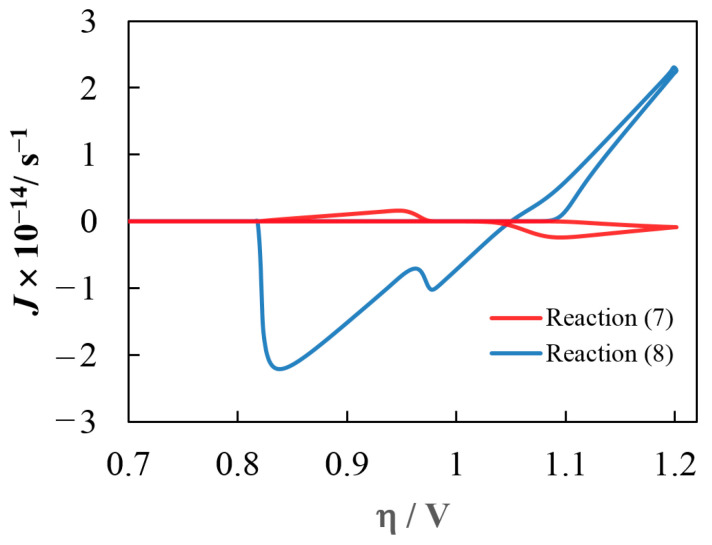
Flows of $W_{n}O_{3n+1}^{2-}$ and $W_{n}O_{3n}^{-}$ due to reactions (7) and (8) resulting in the OTB’s growth/dissolution. The contributions are calculated for one OTB crystal at η_λ_ = 1.20 V and ν = 0.10 V·s^−1^.

The contributions of the electrochemical reaction (7) and chemical reaction (8) to the growth and dissolution of OTB crystals depend on the specific electrodeposition conditions. Figure 9 illustrates the typical contributions of these processes in our case. Under given conditions, in the cathodic region, the growth of Na_x_WO_3_ crystals occurs actually due to the crystal lattice construction by $W_{n}O_{3n}^{-}$ and Na^+^ ions; while reaction (7), which assumes that the electrochemical process happens with the participation of $W_{n}O_{3n+1}^{2-}$ and Na^+^ ions on the OTB, leads to electrodissolution on the surface layer of the crystals. In the anodic region, on the contrary, reaction (7) prevents the dissolution of the inner crystal region.

The change in size (cube edge length) of the Na_x_WO_3_ crystal during the potential scan is shown in Figure 7b,d. It is important to note that both the overpotential at which the formation of stable OTB nuclei begins (about 1.08 V) and the maximum crystal size are in very good agreement with experimental observations. As expected, an increase in the reverse overpotential and a decrease in the scan rate contribute to an increase in the crystal size due to an increase in the time the system remains in the region of higher overpotentials and, accordingly, an increase in the total transferred charge [51,52]. Near the transition to the anodic region, the crystal size is almost constant for some time. The transition from the outer crystal layer’s dissolution to the inner crystal region’s dissolution appears as an inflection in the overpotential range corresponding to the local current maximum between peaks **A′** and **A″**. The values of ζ_0_/ζ_max_ and Δζ/ζ_max_ (see Table 2) show a decrease in the surface layer’s thickness and the transition region’s proportion (see Section 4.3) as the crystal’s growth time increases. Among other results presented in Table 2, we note a good agreement between the experimental and fitting values of the number density of OTB crystals, *N*_OTB_. In addition, the found average value of the diffusion coefficient for $W_{n}O_{3n}^{-}$ (4.43 × 10^−5^ cm^2^s^−1^) is typical for molten salts.

**Figure 10:**
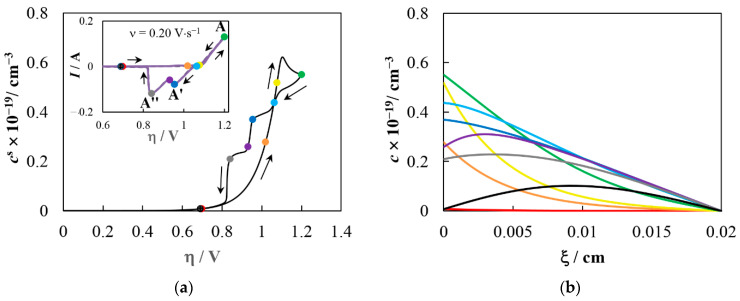
(**a**) Calculated overpotential dependence for the $W_{n}O_{3n}^{-}$ surface concentration corresponding to the cyclic voltammogram in the inset; (**b**) calculated concentration profiles for $W_{n}O_{3n}^{-}$ at several points marked in Figure 10a. The concentration profile color corresponds to the dot color.

Figure 10 shows the calculated dependencies of surface concentration on overpotential (Figure 10a) and concentration profiles (Figure 10b) for $W_{n}O_{3n}^{-}$. During scanning in the cathodic direction, the surface concentration of $W_{n}O_{3n}^{-}$, *c*^s^, first increases exponentially as the overpotential increases due to an increase in the forward reaction (11) rate and a decrease in the reverse reaction (11) rate. The appeared $W_{n}O_{3n}^{-}$ ions together with sodium ions form unstable OTB-like submicrostructures on the electrode surface to which Na^+^, $W_{n}O_{3n}^{-}$ and $W_{n}O_{3n+1}^{2-}$ can attach/detach. Moreover, the rate of $W_{n}O_{3n}^{-}$ attachment depends on the concentration of these ions, and the rate of $W_{n}O_{3n+1}^{2-}$ attachment/detachment depends on the potential. Upon reaching the critical $W_{n}O_{3n}^{-}$ concentration and the electrode potential, stable OTB nuclei are formed whose growth rate exceeds the dissolution rate. Apparently NaW_4_O_12_ may be the smallest stable nucleus. The active consumption of $W_{n}O_{3n}^{-}$ by growing OTB crystals causes a peak in the *c*^s^(η) dependence. After the reversal point (green dot in Figure 10a), the $W_{n}O_{3n}^{-}$ formation rate decreases, and the anodic component of the electrochemical process (7) increases, which leads to a decrease in the OTB growth rate. In the anodic region the deposit dissolves. The stepwise shape of the *c*^s^(η) dependence (see Figure 10a) here is associated with different dissolution rates of the outer crystal layer (between the light blue and blue dots), the intermediate crystal zone (between the blue and violet dots), and the inner crystal region (after the violet dot).

Thus, the proposed approaches allow us to study in detail the mechanism and kinetic patterns of OTB formation/growth/dissolution through simulation and fitting of cyclic voltammograms.

## 6. Conclusions

The formation, growth and dissolution of electrode deposits containing sodium tungsten bronze during the electrolysis of the 0.8Na_2_WO_4_–0.2WO_3_ melt at 1023 K were comprehensively studied.

The experimental results indicate a nontrivial mechanism of the process. To analyze experimental cyclic voltammograms, mathematical models were proposed that consider the growth and dissolution of a two-phase deposit consisting of Na_x_WO_3_ and metallic tungsten or two sodium tungsten bronzes of different compositions. The models describe mass transfer within the diffusion layer to the electrode surface and to the nuclei, as well as the most probable chemical and electrochemical reactions occurring in the melt, on the electrode surface and on the surface of new phases. The processes of the growth/dissolution of nuclei due to electrochemical reactions are modeled for the case of mixed control (diffusion + charge transfer) taking into account the ohmic potential drop.

Moreover, the model was proposed for the dissolution of Na_x_WO_3_ crystals with different sodium content in the surface layer and in the crystal bulk. The use of this model, supplemented by a smooth change in the pre-exponential factors for the backward reactions near the crossover overpotential for the intermediate crystal region, provides the best agreement between the experimental and simulated cyclic voltammograms. This approach allowed us to exclude from the general scheme reactions that weakly affect the current response and to clarify the mechanism of OTB formation.

According to the established mechanism, the formation of OTB crystals is impossible without the electrochemical reduction of tungstate anions by reaction (11), and the OTB growth regularities are determined by the contributions of both the electrochemical process (7) with the participation of $W_{n}O_{3n+1}^{2-}$ and Na^+^, and the chemical reaction (8) between Na^+^ and $W_{n}O_{3n}^{-}$ formed during the $W_{n}O_{3n+1}^{2-}$ electroreduction on the electrode. Under the conditions studied, reaction (8) first promotes the OTB growth (in the cathodic region) and then its dissolution (in the anodic region), while reaction (7) inhibits these processes. The simulation results allowed us to consistently explain all the patterns and characteristic features observed in the experiment.

The proposed approaches and models can be used to analyze the mechanism and kinetics of electrode processes complicated by phase formation, parallel and sequential chemical and electrochemical processes, as well as the dissimilarity of the phase and/or chemical composition of the deposit.

## Figures and Tables

**Table 1:** Elemental analysis at the points marked in Figure 3.

Spectrum	Concentration, at.%			
	O	Na	W	Pt
1	62.02	17.89	20.09	-
2	48.71	16.32	34.97	-
3	7.50	-	-	92.50
4	12.98	-	2.35	84.67
5	59.34	17.61	21.69	1.36
6	8.76	-	0.92	90.32
7	12.14	-	1.30	86.56
8	13.84	-	1.96	84.20
9	51.97	20.25	27.79	-
10	9.51	-	90.49	-
11	42.51	-	57.49	-
12	36.24	-	63.76	-

**Table 2:** Parameter values found by fitting using diagram 8b.

Parameters	ν = 0.1 V·s^–1^			η_λ_ = 1.2 V			Average Value	Standard Deviation
	η_λ_, V			ν, V·s^–1^				
	1.13	1.15	1.20	0.2	0.3	0.4		
$D_{W_{n}O_{3n}^{-}}$, cm^2^s^−1^	3.99 × 10^−5^	4.59 × 10^−5^	4.59 × 10^−5^	4.50 × 10^−5^	4.51 × 10^−5^	4.39 × 10^−5^	4.43 × 10^−5^	2.28 × 10^−6^
$K_{b7}^{c}$, cm·s^–1^	4.59 × 10^15^	5.16 × 10^15^	5.31 × 10^15^	5.14 × 10^15^	5.17 × 10^15^	5.44 × 10^15^	5.13 × 10^15^	2.90 × 10^14^
$K_{b7}^{a}$, cm·s ^–1^	1.55 × 10^20^	4.79 × 10^20^	4.28 × 10^20^	4.71 × 10^20^	5.19 × 10^20^	5.77 × 10^20^	3.88 × 10^20^	1.42 × 10^20^
$K_{f7}^{\mathrm{up}}$, cm·s ^–1^	1.05 × 10^−11^	1.18 × 10^−11^	1.21 × 10^−11^	1.17 × 10^−11^	1.18 × 10^−11^	1.24 × 10^−11^	1.17 × 10^−11^	6.57 × 10^−13^
$K_{f7}^{\mathrm{in}}$, cm·s ^–1^	5.56 × 10^−7^	3.29 × 10^−7^	4.40 × 10^−7^	3.58 × 10^−7^	3.29 × 10^−7^	3.44 × 10^−7^	3.93 × 10^−7^	9.01 × 10^−8^
$K_{b7}^{\mathrm{in}}$, cm·s ^–1^	3.35 × 10^15^	3.76 × 10^15^	3.87 × 10^15^	3.75 × 10^15^	3.77 × 10^15^	3.96 × 10^15^	3.74 × 10^15^	2.11 × 10^14^
$K_{b8}^{c}$, cm·s ^–1^	1.11 × 10^20^	8.94 × 10^19^	8.30 × 10^19^	9.28 × 10^19^	1.05 × 10^20^	1.23 × 10^20^	1.01 × 10^20^	1.50 × 10^19^
$K_{b8}^{a}$, cm·s ^–1^	1.52 × 10^20^	1.15 × 10^20^	1.00 × 10^20^	1.09 × 10^20^	1.26 × 10^20^	1.58 × 10^20^	1.27 × 10^20^	2.34 × 10^19^
$K_{f8}^{\mathrm{up}}$, cm·s ^–1^	28.03	23.98	22.19	22.90	25.75	31.21	25.68	3.43
$K_{f8}^{\mathrm{in}}$, cm·s ^–1^	60.35	83.63	82.19	102.62	93.33	96.97	86.52	15.01
$K_{b8}^{\mathrm{in}}$, cm·s ^–1^	1.22 × 10^20^	1.74 × 10^20^	2.03 × 10^20^	2.51 × 10^20^	2.12 × 10^20^	1.99 × 10^20^	1.94 × 10^20^	4.32 × 10^19^
$k_{0\;9}$, cm^−2^s^−1^	2.34 × 10^15^	2.36 × 10^15^	2.18 × 10^15^	2.35 × 10^15^	2.45 × 10^15^	2.47 × 10^15^	2.36 × 10^15^	1.04 × 10^14^
ζ_0_/ζ_max_	0.684	0.821	0.932	0.862	0.744	0.660	–	–
Δζ/ζ_max_	0.214	0.090	0.044	0.099	0.137	0.178	–	–
*N*_OTB_, cm^−2^	5.04 × 10^3^	3.66 × 10^3^	3.47 × 10^3^	6.13 × 10^3^	8.41 × 10^3^	1.58 × 10^4^	–	–

## Data Availability

Data are contained within the article and Supplementary Materials.

## Supplementary Materials

The following supporting information can be downloaded at: https://www.mdpi.com/article/10.3390/ma16227207/s1.

## References

[1] Labbe P. (1992). Tungsten oxides, tungsten bronzes and tungsten bronze-type structures. Key Eng. Mater..

[2] Bartha L., Kiss A.B., Szalay T. (1995). Chemistry of Tungsten Oxide Bronzes. Int. J. Refract. Met. Hard Mater..

[3] Lawrence S., Stevenson S., Mavadia K., Sermon P. (1987). Solid-State Properties of Some Polycrystalline Alkali-Metal Tungsten Bronzes. Proc. R. Soc. Lond. Ser. A Math. Phys. Sci..

[4] Shanks H.R., Sidles P.H., Danielson G.C., Ward R. (1963). Electrical Properties of the Tungsten Bronzes. Nonstoichiometric Compounds.

[5] Ellerbeck L.D., Shanks H.R., Sidles P.H., Danielson G.C. (1961). Electrical Resistivity of Cubic Sodium Tungsten Bronze. J. Chem. Phys..

[6] Lekshmi I.C., Hegde M.S. (2005). Synthesis and electrical properties of cubic Na_x_WO_3_ thin films across the metal–insulator transition. Mater. Res. Bull..

[7] Raj S., Matsui H., Souma S., Sato T., Takahashi T., Chakraborty A., Sarma D.D., Mahadevan P., Oishi S., McCarroll W.H. (2007). Electronic structure of sodium tungsten bronzes Na_x_WO_3_ by high-resolution angle-resolved photoemission spectroscopy. Phys. Rev. B.

[8] Sano K., Nitta Y., Ōno Y. (2020). Transition Temperature of Superconductivity in Sodium Tungsten Bronze—Theoretical Study Based on First-principles Calculations. J. Phys. Soc. Jpn..

[9] Cogan S.F., Plante T.D., Parker M.A., Rauh R.D. (1986). Free-electron electrochromic modulation in crystalline Li_x_WO_3_. J. Appl. Phys..

[10] Tegg L., Haberfehlner G., Kothleitner G., Kisi E., Keast V.J. (2021). Crystal structures, electrical properties, and electron energy-loss spectroscopy of the sodium and potassium tetragonal tungsten bronzes. J. Alloys Compd..

[11] El-Sayed A.M., Mousa S.M.A. (2005). Some properties of sodium tungsten bronzes as a function of sodium concentration. Ind. J. Chem. Technol..

[12] Wechter M.A., Shanks H.R., Carter G., Ebert G.M., Guglielmino R., Voigt A.F. (1972). Use of metal tungsten bronze electrodes in chemical analysis. Anal. Chem..

[13] Hahn P.B., Wechter M.A., Johnson D.C., Voigt A.F. (1973). Sodium tungsten bronze as a potentiometric indicating electrode for dissolved oxygen in aqueous solution. Anal. Chem..

[14] Griffith C.S., Luca V., Hanna J.V., Pike K.J., Smith M.E., Thorogood G.S. (2009). Microcrystalline Hexagonal Tungsten Bronze. 1. Basis of Ion Exchange Selectivity for Cesium and Strontium. Inorg. Chem..

[15] Egorin A.M., Dran’kov A.N., Didenko N.V., Tokar’ E.A., Sokol’nitskaya T.A., Papynov E.K., Tananaev I.G. (2020). Synthesis and sorption characteristics of tungsten oxides-based materials for Sr-90 removal from water media. J. Mater. Sci..

[16] Zimmer A., Gilliot M., Tresse M., Broch L., Tillous K.E., Boulanger C., Stein N., Horwat D. (2019). Coloration mechanism of electrochromic Na_x_WO_3_ thin films. Opt. Lett..

[17] Tegg L., Cuskelly D., Keast V.J. (2017). The sodium tungsten bronzes as plasmonic materials: Fabrication, calculation and characterization. Mater. Res. Express.

[18] Tegg L., Cuskelly D., Keast V.J. (2018). Plasmon Responses in the Sodium Tungsten Bronzes. Plasmonics.

[19] Azimirad R., Khademi A., Akhavan J., Moshfegh A.Z. (2009). Growth of Na_0.3_WO_3_ nanorods for the field emission application. J. Phys. D Appl. Phys..

[20] Guo C., Yin S., Sato T. (2012). Effects of Crystallization Atmospheres on the Near-Infrared Absorbtion and Electroconductive Properties of Tungsten Bronze Type M_x_WO_3_ (M = Na, K). J. Am. Ceram. Soc..

[21] Kotvanova M.K., Pavlova S.S., Efremova N.N. (2013). Nanosized crystals of oxide bronzes of titanium, molybdenum, tungsten as components of anticorrosive coatings. Izvestiya Vuzov. Khimiya Khimicheskaya Tekhnologiya.

[22] Jie S., Guo X., Ouyang Z. (2019). Tumor ablation using novel photothermal Na_x_WO_3_ nanoparticles against breast cancer osteolytic bone metastasis. Int. J. Nanomed..

[23] Wang L., Zhan J., Fan W., Cui G., Sun H., Zhuo L., Zhao X., Tang B. (2010). Microcrystalline sodium tungsten bronze nanowire bundles as efficient visible light-responsive photocatalysts. Chem. Commun..

[24] Wu C.-M., Naseem S., Chou M.-H., Wang J.-H., Jian Y.-Q. (2019). Recent Advances in Tungsten-Oxide-Based Materials and Their Applications. Front. Mater..

[25] Yi L., Zhao W., Huang Y., Wu X., Wang J., Zhang G. (2020). Tungsten bronze Cs_0.33_WO_3_ nanorods modified by molybdenum for improved photocatalytic CO_2_ reduction directly from air. Sci. China Mater..

[26] Vakarin S.V., Melyaeva A.A., Semerikova O.L., Kondratuk V.S., Pankratov A.A., Plaksin S.V., Porotnikova N.M., Zaykov Y.P., Petrov L.A., Mikushina Y.V. (2011). Catalase activity of coarse grained and nanosized oxide tungsten bronzes obtained by electrolysis of molten salts. Russ. Chem. Bull..

[27] Semerikova O.L., Vakarin S.V., Kosov A.V., Plaksin S.V., Pankratov A.A., Grishenkova O.V., Zaykov Y.P., Shishmakov A.B., Mikushina Y.V., Petrov L.A. (2019). Electrochemical Synthesis of Nanohybrid Systems Based on Copper and the Oxide Tungsten Bronzes. J. Electrochem. Soc..

[28] Drobasheva T.I., Spitsyn V.I., Spitsyn V.I. (1982). Tungsten and molybdenum bronzes with two alkaline elements. Oxide Bronzes.

[29] Kaliev K.A., Baraboshkin A.N., Spitsyn V.I. (1982). Electrocrystallization of transition metal oxide bronzes from molten salts. Oxide Bronzes.

[30] Banks E., Fleischmann C.W., Meites L. (1970). On the nature of the species reduced during the electrochemical synthesis of tungsten bronzes. J. Solid State Chem..

[31] Meites L., Banks E., Fleischmann C.W. (1972). Voltammetric behaviors of platinum electrodes and decomposition potentials of alkali tungstate and polytungstate melts. Anal. Chem..

[32] Fredlein R.A., Damjanovic A. (1972). Electrochemical deposition and dissolution of tungsten oxide bronzes. J. Solid State Chem..

[33] Randin J.-P. (1973). Electrochemical deposition of sodium tungsten bronzes. J. Electrochem. Soc..

[34] Randin J.-P. (1974). Kinetics of the electrochemical deposition and dissolution of sodium tungsten bronzes. Electrochim. Acta.

[35] Elwell D., DeMattei R.C., Zubeck I.V., Feigelson R.S., Huggins R.A. (1976). DC resistances in electrolytic crystallization from molten salts. J. Cryst. Growth.

[36] Okada K., Miyake M., Iwai S., Ohno H., Furukawa K. (1978). Structural Analysis of Molten Na_2_WO_4_. J. Chem. Soc. Faraday Trans. 2.

[37] Miyake M., Okada K., Iwai S., Ohno H., Furukawa K. (1978). Structural analysis of molten Na_2_W_2_O_7_. J. Chem. Soc. Faraday Trans. 2.

[38] Baraboshkin A.N., Kaliev K.A., Aksentiev A.G. (1978). Formation of metastable phases on the cathode in the initial period of electrolysis. Elektrokhimiya.

[39] Afonichkin V.K., Leontiev V.N., Komarov V.E. (1993). Equilibrium electrode potentials of tungsten in melts of the Na_2_WO_4_–WO_3_ system. Elektrokhimiya.

[40] Vorozhbit V.U., Shurdumov G.K., Kaliev K.A. (1995). Obtaining highly dispersed powders of oxide lithium-tungsten bronzes. Tsvetnye Met..

[41] Voron’ko Y.K., Sobol’ A.A. (2005). Influence of Cations on the Vibrational Spectra and Structure of [WO_4_] Complexes in Molten Tungstates. Inorg. Mater..

[42] Voronko Y.K., Sobol A.A., Shukshin V.E. (2014). Raman scattering study of molten alkali-metal molybdates and tungstates rich in basic oxides. Inorg. Mater..

[43] Wang J., You J.L., Wang Y.Y., Wang M., Jun W.U. (2016). In-situ Raman Spectroscopic Study of the Molten Tungstates in Li_2_O-WO_3_ Binary System. Chin. J. Light Scatt..

[44] Wang J., You J., Wang M., Lu L., Wan S., Sobol A.A. (2017). In-situ studies on the micro-structure evolution of A_2_W_2_O_7_ (A = Li, Na, K) during melting by high temperature Raman spectroscopy and density functional theory. Spectrochim. Acta A.

[45] Wang J., You J.L., Sobol A.A., Lu L.M., Wang M., Wu J., Lv X.M., Wan S.M. (2017). In-situ high temperature Raman spectroscopic study on the structural evolution of Na_2_W_2_O_7_ from the crystalline to molten states. J. Raman Spectrosc..

[46] Kosov A.V., Semerikova O.L., Vakarin S.V., Grishenkova O.V., Vorob’ev A.S., Khudorozhkova A.O., Zaikov Y.P. (2022). Ionic Equilibria in Polytungstate Melts. Processes.

[47] Baraboshkin A.N., Tarasova K.P., Nazarov B.A., Martemyanova Z.S. (1973). Study of the composition and structure of cathode deposits during the electrolysis of molten Na_2_WO_4_-WO_3_ mixtures. Electrochem. Molten Salt Solid Electrolytes Proc. Inst. Electrochem. AS USSR.

[48] Fletcher S., Halliday C.S., Gates D., Westcott M., Lwin T., Nelson G. (1983). The response of some nucleation/growth processes to triangular scans of potential. J. Electroanal. Chem..

[49] Velmurugan J., Noël J.-M., Nogala W., Mirkin M.V. (2012). Nucleation and growth of metal on nanoelectrodes. Chem. Sci..

[50] Isaev V.A., Grishenkova O.V., Zaykov Y.P. (2018). Theory of cyclic voltammetry for electrochemical nucleation and growth. J. Solid State Electrochem..

[51] Kosov A.V., Grishenkova O.V., Semerikova O.L., Isaev V.A., Zaykov Y.P. (2021). On the theory of cyclic voltammetry for multiple nucleation and growth: Scan rate influence. J. Electroanal. Chem..

[52] Grishenkova O.V., Kosov A.V., Semerikova O.L., Isaev V.A., Zaikov Y.P. (2021). Theoretical and experimental cyclic voltammetry studies of the initial stages of electrocrystallization. Russ. Metall. (Met.).

[53] Scharifker B.R., Hills G.J. (1983). Theoretical and experimental studies of multiple nucleation. Electrochim. Acta.

[54] Isaev V.A., Grishenkova O.V., Zaykov Y.P. (2016). Analysis of the geometrical–probabilistic models of electrocrystallization. Russ. Metall. (Met.).

[55] Isaev V.A., Zaykov Y.P., Grishenkova O.V., Kosov A.V., Semerikova O.L. (2019). Analysis of Potentiostatic Current Transients for Multiple Nucleation with Diffusion and Kinetic Controlled Growth. J. Electrochem. Soc..

[56] Scharifker B.R., Mostany J. (1984). Three-dimensional nucleation with diffusion controlled growth. Part I. Number density of active sites and nucleation rates per site. J. Electroanal. Chem..

[57] Abyaneh M.Y. (1991). Formulation of a general model for nucleation and growth of electrodeposits. Electrochim. Acta.

[58] Altimari P., Pagnanelli F. (2016). Electrochemical nucleation and three-dimensional growth under mixed kinetic-diffusion control: Analytical approximation of the current transient. Electrochim. Acta.

[59] Milchev A., Zapryanova T. (2006). Nucleation and growth of copper under combined charge transfer and diffusion limitations: Part I. Electrochim. Acta.

[60] Milchev A., Zapryanova T. (2006). Nucleation and growth of copper under combined charge transfer and diffusion limitations: Part II. Electrochim. Acta.

[61] Kosov A.V., Grishenkova O.V., Isaev V.A., Zaikov Y. (2022). Simulation of Diffusion-Controlled Growth of Interdependent Nuclei under Potentiostatic Conditions. Materials.

[62] Isaev V.A., Grishenkova O.V., Kosov A.V., Semerikova O.L., Zaikov Y. (2021). Simulation of 3D electrochemical phase formation: Mixed growth control. Materials.

[63] Kosov A.V., Grishenkova O.V. (2023). Modeling and simulation of 3D electrochemical phase formation under mixed kinetic-diffusion growth control. J. Solid State Electrochem..

[64] D’Ajello P.C.T., Munford M.L., Pasa A.A. (1999). Transient equations for multiple nucleation on solid electrodes: A stochastic description. J. Chem. Phys..

[65] D’Ajello P.C.T., Fiori M.A., Pasa A.A., Kipervaser Z.G. (2000). Reaction-Diffusion Interplay in Electrochemical Deposition Processes. A Theoretical Approach. J. Electrochem. Soc..

[66] Luo G., Li D., Yuan G., Li N. (2018). Potentiostatic Current Transient for Multiple Nucleation: A Limited-Diffusion Process Description. J. Electrochem. Soc..

[67] Volgin V.M., Volgina O.V., Davydov A.D. (2003). Finite difference method of simulation of non-steady-state ion transfer in electrochemical systems with allowance for migration. Comput. Biol. Chem..

[68] Myland J.C., Oldham K.B. (2002). Convolutive modelling in the absence of supporting electrolyte: Coping with migration and changing resistance in predicting voltammetry. J. Electroanal. Chem..

[69] Chadwick A.F., Vardar G., DeWitt S., Sleightholme A.E.S., Monroe C.W., Siegel D.J., Thornton K. (2016). Computational Model of Magnesium Deposition and Dissolution for Property Determination via Cyclic Voltammetry. J. Electrochem. Soc..

[70] Isaev V.A. (2007). Electrochemical Phase Formation.

[71] Galus Z. (1994). Fundamentals of Electrochemical Analysis.

[72] Bard A.J., Faulkner L.R. (2001). Electrochemical Methods: Fundamentals and Applications.

[73] Brown B.W., Banks E. (1954). The Sodium Tungsten Bronzes. J. Am. Chem. Soc..

[74] Walkenbach J. (2013). Excel 2013 Power Programming with VBA.

[75] Metso HSC Chemistry. www.outotec.com/HSC.

