# Supplementary material for: Mechanism and Kinetics of the Phase Formation and Dissolution of NaxWO3 on a Pt Electrode in a Na2WO4–WO3 Melt
Source: Materials (Basel). 2023 Nov 17;16(22):7207. doi: 10.3390/ma16227207 (PMC10672828; doi:10.3390/ma16227207)
Supplement: Supplementary file 1 [file materials-16-07207-s001.zip › materials-2713218-supplementary.pdf]

Supplementary materials

Na_xWO_3 structure type

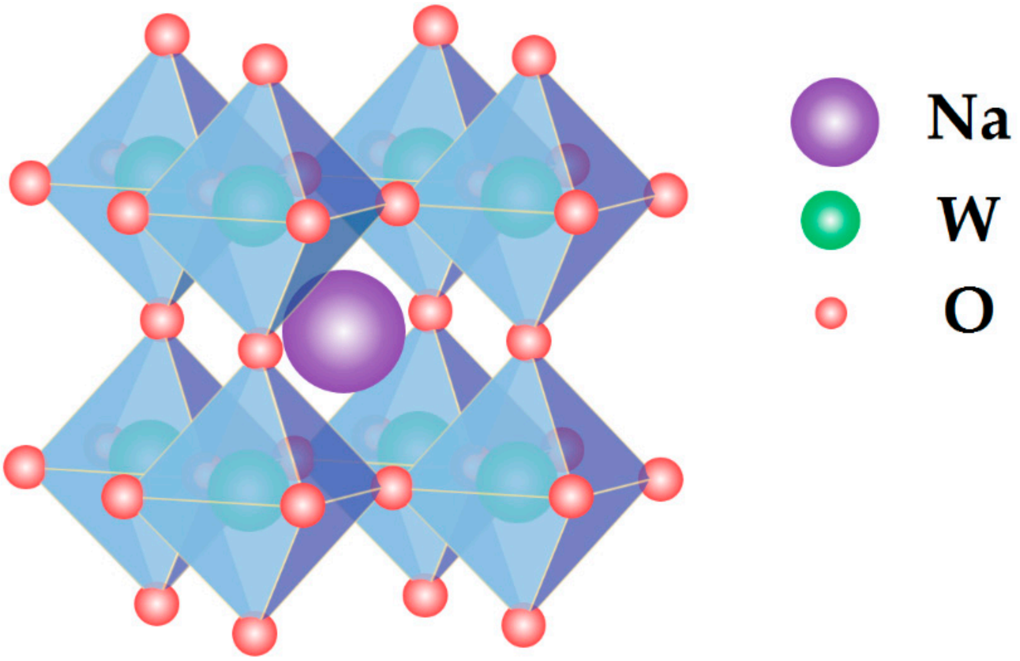

Figure S1. Model of the crystal lattice of cubic sodium tungsten bronze. ABO_3 crystal lattice type (perovskite).

SEM

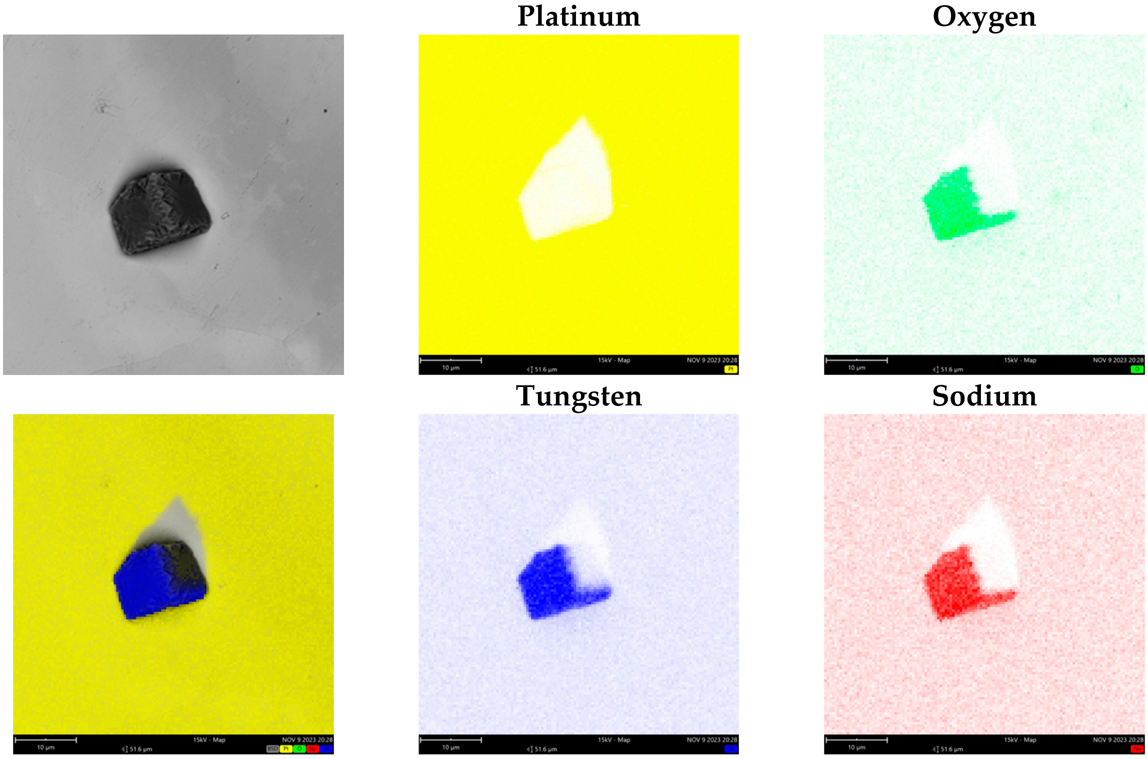

Figure S2. SEM image and element maps for the cubic OTB crystal on the Pt surface. The sample was obtained during the potentiostatic electrolysis of the $0.8Na_2WO_4$–$0.2WO_3$ melt at 1023 K and $E = -1.05$ V for $t = 5$ s.

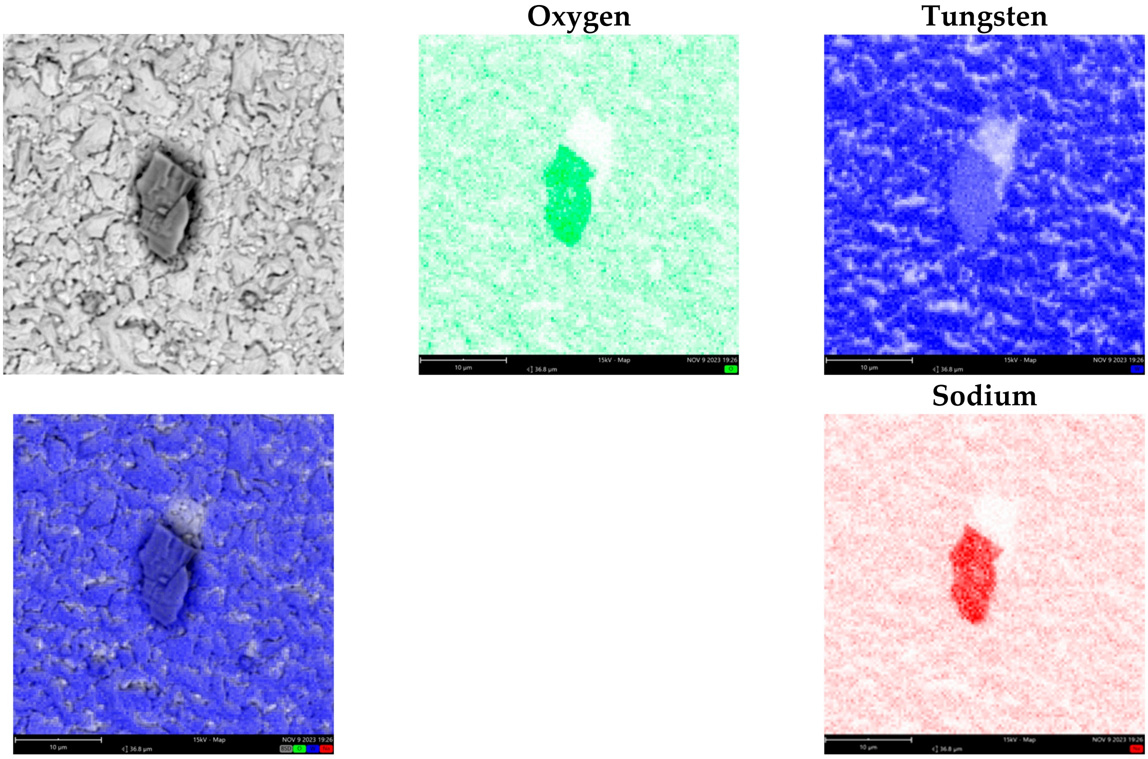

Figure S3. SEM image and element maps for the cubic OTB crystal on the tungsten layer. The sample was obtained during the potentiostatic electrolysis of the $0.8Na_2WO_4$–$0.2WO_3$ melt at 1023 K and $E = -1.20$ V for $t = 60$ s.

XRD patterns

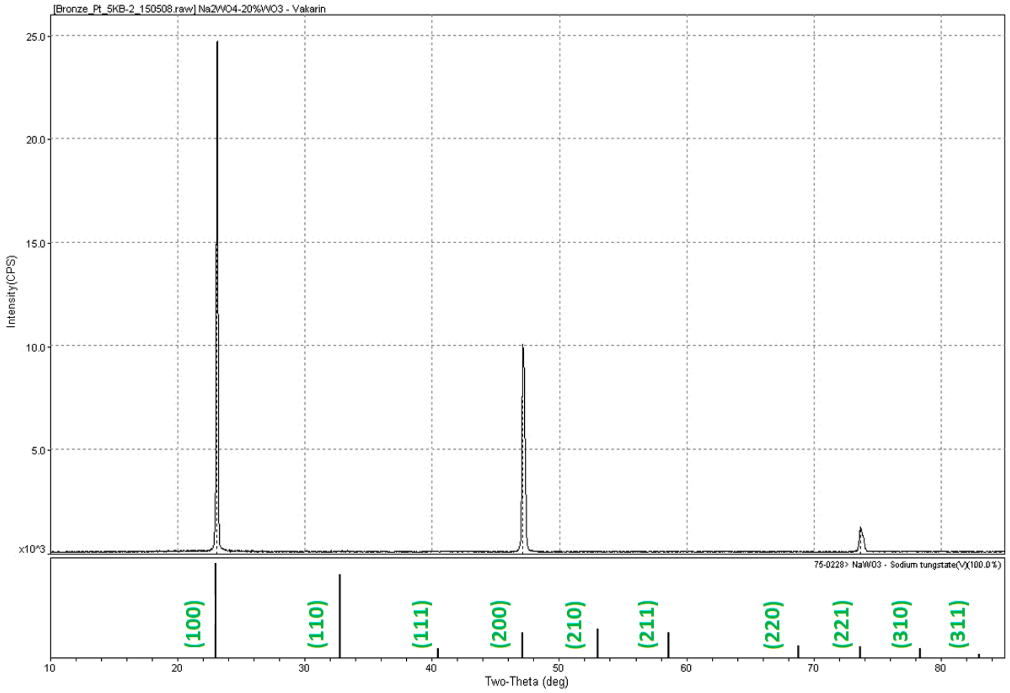

Figure S4. XRD pattern for OTB crystals formed during the electrolysis of the $0.8Na_2WO_4$–$0.2WO_3$ melt at $T = 1023$ K and $E = -1.05$ V for 15 s. The average crystallite size in the $NaWO_3$ crystal calculated using the Eq. (S1) is $d = 1.00 \pm 0.03$ nm.

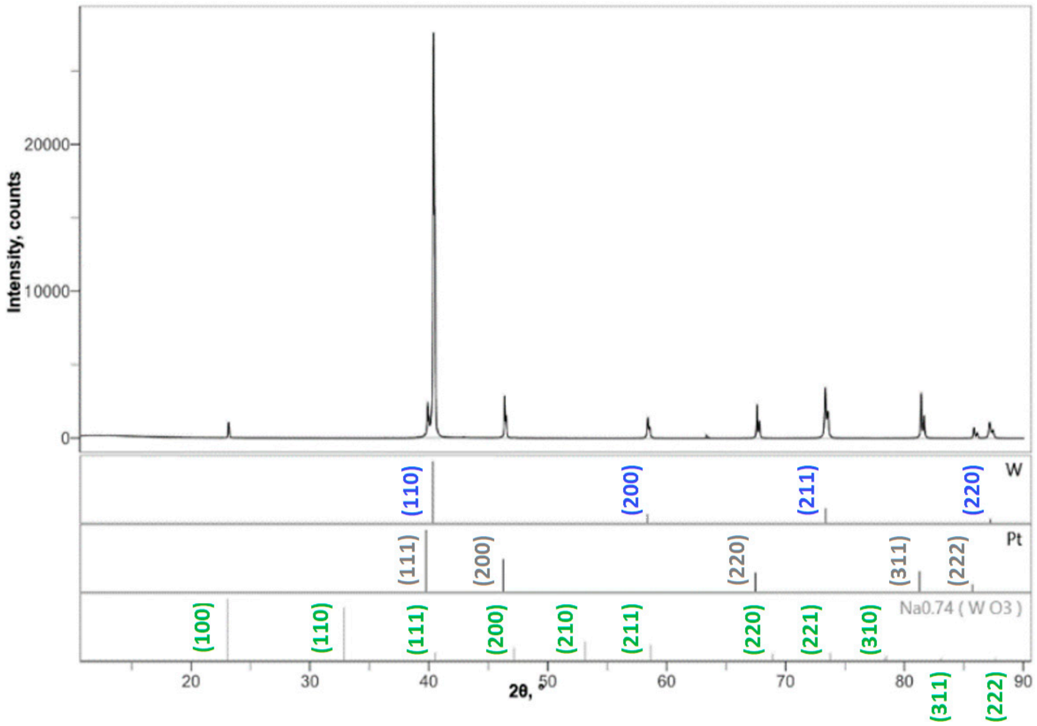

Figure S5. XRD pattern for deposit formed on the Pt substrate during the electrolysis of the $0.8Na_2WO_4$–$0.2WO_3$ melt at $T = 1023$ K and $E = -1.20$ V for $t = 60$ s. The average crystallite size in the $Na_{0.74}WO_3$ crystal calculated using the Eq. (S1) is $d = 1.54 \pm 0.16$ nm.

To calculate the average size of coherent scattering regions (domains, crystallites), d, the Scherrer equation can be used [Gusev, A.I. *Nanomaterials, nanostructures, nanotechnologies*, 2nd ed. (in Russian); Science: Moscow, Russia, 2005]:

$$d = K\lambda(\beta\cos\theta)^{-1},\ K = 6|h|^3\,[(h^2+k^2+l^2)^{0.5}(6h^2-2|hk|+|kl|-2|hl|)]^{-1}, \quad \text{(S1)}$$

where K is a dimensionless shape factor, λ is the X-ray wavelength, β is the line broadening at half the maximum intensity, θ is the Bragg angle, and h,k,l are Miller indices. The d values calculated using equation (S1) for the OTB samples are given in the captions to Figures S1 and S2. As can be seen, an increase in the potential and duration of electrolysis contributes to an increase in the d value.
